# Effects of Graded Whey Supplementation During Extreme-Volume Resistance Training

**DOI:** 10.3389/fnut.2018.00084

**Published:** 2018-09-11

**Authors:** Cody T. Haun, Christopher G. Vann, Christopher B. Mobley, Paul A. Roberson, Shelby C. Osburn, Hudson M. Holmes, Petey M. Mumford, Matthew A. Romero, Kaelin C. Young, Jordan R. Moon, L. Bruce Gladden, Robert D. Arnold, Michael A. Israetel, Annie N. Kirby, Michael D. Roberts

**Affiliations:** ^1^School of Kinesiology, Auburn University, Auburn, AL, United States; ^2^Department of Cell Biology and Physiology, Edward Via College of Osteopathic Medicine-Auburn Campus, Auburn, AL, United States; ^3^Impedimed Inc., Carlsbad, CA, United States; ^4^Harrison School of Pharmacy, Auburn University, Auburn, AL, United States; ^5^Renaissance Periodization, Charlotte, NC, United States

**Keywords:** muscle hypertrophy, resistance training, recovery, adaptation, graded whey protein

## Abstract

We examined hypertrophic outcomes of weekly graded whey protein dosing (GWP) vs. whey protein (WP) or maltodextrin (MALTO) dosed once daily during 6 weeks of high-volume resistance training (RT). College-aged resistance-trained males (training age = 5 ± 3 years; mean ± SD) performed 6 weeks of RT wherein frequency was 3 d/week and each session involved 2 upper- and 2 lower-body exercises (10 repetitions/set). Volume increased from 10 sets/exercise (week 1) to 32 sets/exercise (week 6), which is the highest volume investigated in this timeframe. Participants were assigned to WP (25 g/d; *n* = 10), MALTO (30 g/d; *n* = 10), or GWP (25–150 g/d from weeks 1–6; *n* = 11), and supplementation occurred throughout training. Dual-energy x-ray absorptiometry (DXA), vastus lateralis (VL), and biceps brachii ultrasounds for muscle thicknesses, and bioelectrical impedance spectroscopy (BIS) were performed prior to training (PRE) and after weeks 3 (MID) and 6 (POST). VL biopsies were also collected for immunohistochemical staining. The GWP group experienced the greatest PRE to POST reduction in DXA fat mass (FM) (−1.00 kg, *p* < 0.05), and a robust increase in DXA fat- and bone-free mass [termed lean body mass (LBM) throughout] (+2.93 kg, *p* < 0.05). However, the MALTO group also experienced a PRE to POST increase in DXA LBM (+2.35 kg, *p* < 0.05), and the GWP and MALTO groups experienced similar PRE to POST increases in type II muscle fiber cross-sectional area (~+300 μm^2^). When examining the effects of training on LBM increases (ΔLBM) in all participants combined, PRE to MID (+1.34 kg, *p* < 0.001) and MID to POST (+0.85 kg, *p* < 0.001) increases were observed. However, when adjusting ΔLBM for extracellular water (ECW) changes, intending to remove the confounder of edema, a significant increase was observed from PRE to MID (+1.18 kg, *p* < 0.001) but not MID to POST (+0.25 kg; *p* = 0.131). Based upon DXA data, GWP supplementation may be a viable strategy to improve body composition during high-volume RT. However, large LBM increases observed in the MALTO group preclude us from suggesting that GWP supplementation is clearly superior in facilitating skeletal muscle hypertrophy. With regard to the implemented RT program, ECW-corrected ΔLBM gains were largely dampened, but still positive, in resistance-trained participants when RT exceeded ~20 sets/exercise/wk.

## Introduction

Resistance training (RT) is well documented to enhance skeletal muscle hypertrophy, and greater RT volume (e.g., 1 set vs. 3 sets) is associated with higher muscle protein turnover (1). Numerous studies indicate post-exercise protein ingestion, particularly whey protein, acutely stimulates significant increases in post-exercise muscle protein synthesis (MPS) [reviewed in (2)]. Moreover, significantly greater acute post-exercise MPS responses have been shown to occur with the ingestion of moderate whey protein doses (≥35 g) compared to lower doses (e.g., ≤20 g) (3, 4). It has been argued that the consumption of very high protein doses (e.g., 60+ g) do not further stimulate post-exercise MPS levels relative to moderate doses (e.g., 30–40 g). For instance, recent meta-analytical data from Morton et al. (5) suggests a plateau in hypertrophic benefits of protein intake when combined with RT beyond doses of ~1.60 g/kg/day based on data from 49 studies with 1,863 participants combined. Furthermore, Moore et al. (6) reported via breakpoint analysis that protein doses of ~0.30 g/kg maximally stimulated myofibrillar fractional synthesis rates at rest. However, considering that greater RT volumes induce higher rates of muscle protein turnover (7), a potential confounding variable in the analysis by Morton et al. is the heterogeneity in RT volume completed in the studies analyzed. Additionally, the analysis by Moore et al. was completed on data derived from resting subjects whom consumed varying doses of protein and not based on data derived from subjects consuming protein after RT. As argued by Wolfe (8), the highest net muscle protein balances have been observed after both RT and ingestion of protein compared to one or the other. Moreover, higher RT volumes may increase the need for protein to optimize the hypertrophic response.

Indeed, evidence suggests high-dose whey protein supplementation combined with supervised RT enhances skeletal muscle hypertrophy. For instance, four studies in previously-trained subjects have reported that high-dose (~80–120 g/d) supplementation with whey protein (or a protein blend containing whey protein) significantly increases fat free mass following 6–12 weeks of RT (9–12). However, Lockwood et al. (13) reported that 60 g/d of whey protein concentrate or hydrolyzed whey protein supplementation over an 8-week period did not further increase fat free mass in previously-trained subjects compared to counterparts supplementing with maltodextrin. Along with the above, two additional lines of evidence suggest that graded intakes of whey protein concurrent to graded increases in RT volume could enhance a short-term hypertrophic response to RT. As stated previously, Burd et al. (1) reported significantly higher MPS rates after 3 sets of leg extensions compared to 1 set of leg extensions with the same relative load, indicating that higher volumes of RT result in greater acute increases in MPS, at least to a point. This acute data agrees with recent meta-analytical data from Schoenfeld et al. (14) suggesting greater hypertrophy in response to 10 or more sets of RT per muscle per week compared to 5 or less sets.

It stands to reason that concurrently increasing the dosage of whey protein consumption and RT volume could enhance short-term muscle hypertrophy given that: (a) there have been observed increases in MPS in response to graded amounts of whey protein consumption, and (b) higher protein intakes as well as higher RT volumes generally result in greater hypertrophic outcomes. However, no studies to date have investigated if incrementally dosing whey protein in a proportional manner to RT volume is a viable strategy for enhancing skeletal muscle hypertrophy in well-trained subjects. Therefore, the purpose of this study was to investigate the potential hypertrophic effects of graded whey protein supplementation dosing during unaccustomed and extremely voluminous RT. To accomplish this aim, participants in this study were instructed to consume either: (a) a single 25 g supplemental dose of whey protein per day (WP), (b) a graded dose of protein throughout the study for which the dose per day was increased by 25 g each week [GWP (25–150 g from week 1 to week 6)], or (c) a single 30 g supplemental dose of a maltodextrin-based carbohydrate supplement per day (MALTO). As a secondary aim, we sought to examine the effects of RT volumes higher than previously investigated during a 6-week timeframe on hypertrophic outcomes in all participants independent of supplementation. Given the exploratory nature of this work, we adopted a null hypothesis for all independent and dependent variable relationships.

## Materials and methods

### Ethical approval and participant screening

This study was approved by the Institutional Review Board at Auburn University and conformed to the standards set by the latest revision of the Declaration of Helsinki (IRB approval #: 17-425 MR 1710). Resistance-trained young men from the local community were recruited to participate in this study. Participants provided both verbal and written informed consent, and completed a medical history form prior to screening. Two primary criteria were used to establish training status: (a) self-reported >1.0 years of RT, and (b) back squat 1RM ≥1.5 × body mass [estimated from a three-repetition maximum (3RM) test conducted for each participant with strict criteria (e.g., crease of the hip below the top of the knee joint at the bottom of the squat)]. After screening, 34 participants were counterbalanced among groups to ensure no significant differences existed between groups in DXA fat- and bone-free mass (termed lean body mass [LBM] throughout) and 3RM squat at baseline. One participant withdrew from the study during week 1 for personal reasons, and 2 others missed more than 3 sessions over the course of the first 4 weeks and we did not feel comfortable with making up this volume during other days/weeks so these participants were removed from the study. Hence, 31 participants completed the study and were partitioned to one of three groups: (1) daily single dose of whey protein (WP, 25 g/d; *n* = 10), (2) daily single dose of maltodextrin (MALTO, 30 g/d; *n* = 10), or (3) graded dose of WP (GWP, 25–150 g/d from weeks 1 to 6; *n* = 11). Descriptive characteristics are provided in Table 1 below and in Supplementary Table 1. Notably, participants were instructed to refrain from ergogenic aids throughout the duration of the study (particularly pre-workout supplements, amino acid, or protein supplements), but were not restricted from using the following (if chronically consumed prior to the study): (a) multivitamin-mineral supplement, (b) creatine monohydrate, or (c) caffeine in the form of coffee.

**Table 1:** Pre-study body composition and strength descriptive measurements.

**Variable**	**WP (*n* = 10)**	**GWP (*n* = 11)**	**MALTO (*n* = 10)**	**Total (*n* = 31)**
Age (years)	21.20 ± 2.39	20.60 ± 1.51	22.10 ± 2.28	21.48 ± 2.13
Height (cm)	177.85 ± 5.60	177.75 ± 9.56	184.00 ± 7.55	179.81 ± 7.91
Weight (kg)	82.19 ± 8.69	84.51 ± 14.34	81.35 ± 10.72	82.74 ± 11.29
Total lean mass (kg)	63.73 ± 7.11	67.15 ± 10.90	62.06 ± 8.44	64.45 ± 9.08
Total fat mass (kg)	15.18 ± 4.15	13.82 ± 4.74	16.10 ± 3.55	14.94 ± 4.12
Squat 3RM (kg)	134.53 ± 21.36	135.70 ± 15.18	126.13 ± 17.25	132.24 ± 17.93
Bench press 3RM (kg)	106.85 ± 19.47	99.82 ± 24.79	89.61 ± 11.43	98.79 ± 20.20
Stiff-legged deadlift 3RM (kg)	129.31 ± 33.00	140.45 ± 28.29	118.42 ± 15.75	129.75 ± 27.43
Lat pulldown 3RM (kg)	74.41 ± 9.85	74.66 ± 17.26	68.51 ± 9.19	72.60 ± 12.73
Overhead press 3RM (kg)	61.25 ± 10.53	55.19 ± 15.97	54.22 ± 5.18	56.83 ± 11.67

*All data presented as means ± standard deviation values*.

### Study design

**Figure 1:**
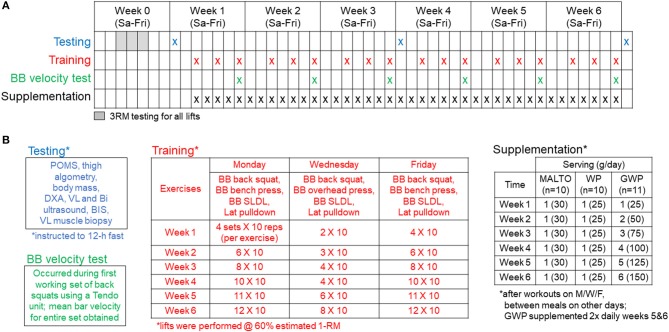
Study design. Panel **(A)** outlines testing, training, and supplementation days. Panel **(B)** (upper left inset) describes the testing battery which included (in order) a profile and mood state questionnaire (POMS), outer thigh pain assessment using algometry, body mass assessment, and whole-body dual x-ray absorptiometry (DXA) scan, a vastus lateralis (VL) and biceps (Bi) ultrasound, total body water assessment using bioelectrical impedance spectroscopy (BIS), and a VL muscle biopsy. Panel **(B)** (lower left inset) describes the BB squat velocity test that occurred during the first set of barbell squats every Friday from weeks 1 to 6 of training. Panel **(B)** (middle inset) outlines the supervised training regimen described in greater detail in the methods. Panel **(B)** (right inset) outlines the supplementation regimen described in greater detail in the methods.

Figure 1 provides a visual representation of the study design. Briefly, a battery of tests was performed prior to week 1 (PRE), after week 3 (MID), and after week 6 (POST). These tests will be further described below following an explanation of the resistance training program, supplementation paradigm, and nutritional recommendations.

### Resistance training

Participants were familiarized with the design of training and technical parameters during testing of 3RMs which occurred 3–7 days prior to PRE testing and training initiation. Strict technical parameters were employed for testing to ensure accurate reflections of strength under direct supervision of research staff holding the Certified Strength and Conditioning Specialist Certification from the National Strength and Conditioning Association.

Following the PRE testing battery and 3RM testing, RT occurred 3 days per week and was progressed according to Figure 1B. Loads corresponding to 60% 1RM, based on 3RM testing, were programmed for each set of each exercise. Sets of 10 repetitions were programmed for each set of each exercise throughout the study. Prior to beginning each training session, participants were instructed to perform a general warm-up involving 25 jumping jacks, 10 bodyweight squats, 10 push-ups, and 10 bodyweight standing reaches mimicking the kinematics of the stiff-legged deadlift (SLDL) for 2 rounds. Next, participants were instructed to perform the following specific warm-up for each exercise: 50% of working set weight for 10 repetitions, 75% for 3 repetitions, and 95–100% for 1 repetition. Exercises were completed one set at a time, in the following order during each training session: Days 1 and 3—barbell (BB) back squat, BB bench press, BB SLDL, and an underhand grip cable machine pulldown exercise designed to target the elbow flexors and latissimus dorsi muscles (Lat Pulldown); Day 2—BB back squat, BB overhead (OH) press, BB SLDL, and Lat Pulldown. A single set of one exercise was completed, followed by a set of each of the succeeding exercises before starting back at the first exercise of the session (e.g., compound sets or rounds). Participants were recommended to take 2 min of rest between each exercise of the compound set. Additionally, participants were recommended to take 2 min of rest between each compound set. However, if participants felt prepared to execute exercises with appropriate technique under investigator supervision they were allowed to proceed to the next exercise without 2 min of rest. Additionally, if participants desired slightly longer than 2 min of rest, this was allowed with intention for the participant to execute the programmed training volume in < 2 h each training session. This design was based on evidence indicating that total volume load (sum of the total repetitions x weight for each individual exercise) for a week of training is primarily related to hypertrophic outcomes, with specific rest intervals between sets being less important. In the interest of ecological validity, we elected a more self-regulated pace of the training session in which participants could be somewhat autonomous while under direct supervision of research staff ensuring technical execution of exercises. Both the extremely high training volumes planned for this investigation and pilot testing of this design led to the implementation of this rest scheme paradigm.

During training sessions, participants provided a repetition in reserve (RIR) rating after each set of each exercise to a researcher, having been instructed to provide a number of repetitions the participant felt he could have completed with good technique beyond the 10 repetitions completed for the set. If the execution of repetitions during a working set was deemed unsafe, or the participant felt unsafe or too fatigued to continue the set or the session, the set or session was terminated. This occurred on only a few occasions, and if repetitions were missed, attempts were made to make these up within the same week of training. The number of repetitions completed for each exercise and the load used for each exercise each week were recorded in Google Sheets (Mountain View, CA, USA), along with the RIR rating provided by the participant for each individual set. RT volume and RIR data are available in the supplementary .csv file (Supplementary SDC 1). Based on pilot testing, we elected a systematic approach to load manipulation within each training session; the load was decreased by 5% for each repetition below 10 (e.g., 9 repetitions = −5%, 8 repetitions = −10%, 7 repetitions = −15%, etc.). However, this was only necessary on a few occasions, and the majority of the training was executed according to the planned study design. BB velocity was also measured using a Tendo unit (TENDO Sports Machines, Trencin, Slovak Republic) on Friday of each week as a proxy of fatigue status and recovery on the first set of BB back squats similar to the methods of Zourdos et al. (15). However, due to logistical constraints, BB velocity was only obtained from a subset of participants at all sampling times (*n* = 6–7 per group). Finally, participants were allowed to train from either 07:00 to 09:00 or 15:30 to 18:30 on Monday, Wednesday, and Friday of each week, and were instructed to perform no other vigorous exercise outside of the study.

### Supplementation

As illustrated in Figure 1, participants were assigned to either MALTO, WP, or GWP groups. All supplements were donated by Dymatize Nutrition® (Dallas, TX, USA). Packaging and delivery was designed to blind participants to the supplement condition; however, investigators of the study were not blinded. The WP (Elite 100% Whey) was comprised of the following nutrition profile per scoop: calories−140, total fat−2 g, cholesterol−70 mg, sodium−70 mg, potassium−150 mg, total carbohydrate−3 g, protein−25 g. Additionally, one WP scoop contained 5.5 g branched chain amino acids (2.7 g L-leucine, 1.4 g L-isoleucine, 1.4 g L-valine), 6.3 g of other essential amino acids, 4.4 g of L-glutamine, 2.4 g of conditionally essential amino acids, and 6.5 g of non-essential amino acids. The MALTO supplement contained 120 calories from 30 g of maltodextrin powder (~30 g of carbohydrates) with <1 g of vanilla flavoring.

Drinks were formulated by research staff for each participant by combining the appropriate serving size with ~500 ml of tap water, and participants consumed drinks after each training session under investigator supervision. The MALTO and WP groups consumed a single scoop each day for the duration of the study; specifically, 1 after training sessions on training days and 1 between meals on non-training days which participants prepared themselves. The GWP group consumed the protein supplement according to the following dosage and timing breakdown:

Week 1: 1 scoop with 500 ml of water post-training on training days, 1 scoop with 500 ml of water between meals on non-training days (1 total scoop each day)
Week 2: 2 scoops with 500 ml of water post-training on training days, 2 scoops with 500 ml of water between meals on non-training days (2 total scoops each day)
Week 3: 3 scoops with 500 ml of water post-training on training days, 3 scoops with 500 ml of water between meals on non-training days (3 total scoops each day)
Week 4: 4 scoops with 500 ml of water post-training on training days, 4 scoops with 500 ml of water between meals on non-training days (4 total scoops each day)
Week 5: 4 scoops with 500 ml of water post-training on training days, 4 scoops with 500 ml of water between meals on non-training days, 1 scoop prior to bed each day (5 total scoops each day)
Week 6: 4 scoops with 500 ml of water post-training on training days, 4 scoops with 500 ml of water between meals on non-training days, 2 scoops prior to bed each day (6 total scoops each day)

Relative supplemental whey intake for the WP group was ~0.30 g/kg/d (i.e., 25 grams of whey/~87 kg body mass). The most the GWP subjects consumed at once post-workout was 100 g. Therefore, the relative intake values for each week post-workout based on the average body mass of the GWP subjects was as follows: 0.30 g/kg/d for week 1, 0.59 g/kg/d for week 2, 0.89 g/kg/d for week 3, and 1.18 g/kg/d for weeks 4–6. As stated above, the remaining GWP doses for weeks 5 and 6 (i.e., 25 additional grams during week 5 and 50 additional grams for week 6) were instructed to be consumed either between meals or before bed. Thus, total supplemental whey values for weeks 5 and 6 were 1.48 g/kg/d and 1.77 g/kg/d, respectively. Beyond post-exercise supplementation which was supervised, participants from all groups verbally reported compliance to the supplementation paradigm on a weekly basis to the research staff. Additionally, participants were asked to refrain from the use of other protein supplements or protein bars throughout the duration of the study.

### Nutritional recommendations and monitoring throughout the protocol

In collaboration with a Registered Dietitian (AK., Ph.D., RD), participants were provided with calorie and macronutrient recommendations along with lists of potential food choices to help meet recommendations for each day during the study. Specifically, recommended values and calculations can be found in the supplementary .csv file (Supplementary SDC 2). These recommendations were based on the following: (a) resting metabolic rate estimates from the Harris-Benedict equation, (b) an estimated non-exercise activity expenditure in this age cohort, (c) an estimated energy expenditure from training each week, and (d) the desire for participants to be in a modest calorie surplus [~500 calories above the estimated total daily energy expenditure (TDEE)] throughout the study. Calculations and supplementary formulae can be found in the supplementary .csv file (Supplementary SDC 2). Additionally, recommendations were provided directly to participants through Google Sheets. Participants were asked to enter dietary intakes each day throughout the study, and include the consumption of their supplement in their daily tracking using a mobile application (MyFitnessPal, Inc.; Baltimore, MD, USA). This mobile application has been validated against paper-based food records (16). Data were exported on a weekly basis for analysis. A de-identified generic food item was created in the application's database for WP, and participants were instructed to log this food item each time a single scoop of their respective supplement was consumed. Entries by participants in the MALTO group were corrected following the study to account for macronutrient differences between the WP and MALTO supplements.

During week 1, participants in the WP and GWP groups were instructed to consume the same daily amount of dietary protein (1.6 g/kg/day) assuming the consumed supplement contributed 25 g/scoop to this total. Participants in the MALTO group were also instructed to consume 1.6 g/kg/day protein during the entire 6-week protocol. This recommendation was based on the findings of Morton et al. (5) suggesting a maximum effective dose of daily protein around this value in young, resistance-trained men. Following week 1, the WP and GWP participants were recommended to increase protein intakes by 25 g per week. However, the GWP group accomplished this increase through supplemental whey protein, whereas the WP group were recommended to consume more protein-rich food sources. Hence, there was no difference in protein dose recommendations between the WP and GWP groups, but the GWP group was expected to obtain more protein on a weekly basis through supplementation. All participants were instructed to consume ~3 g/kg/day of dietary carbohydrate starting on week 1 of the study. A modest amount of carbohydrates (~30 g) were added to this value on training days each week to account for potential reductions in muscle glycogen from increases in training volume based on the recommendations from Scott et al. (17). Fat recommendations were based on remaining calorie values upon setting targeted protein and carbohydrate values. Participants were instructed to attempt to meet the dietary fat recommendation through primarily monounsaturated and polyunsaturated fatty acid sources, while confining saturated fat intakes to no more than 10% of total calorie intake. Logged nutrition data were stored in Google Drive and are provided in a supplementary file in .csv format (Supplementary SDC 1).

### Testing battery procedures

As outlined in Figure 1, the following tests were performed prior to (PRE), during (MID), and following the 6-week protocol (POST). Participants were encouraged to arrive to these testing sessions in an overnight fasted condition approximately 24 h after the third training session during week 3 (for MID) and week 6 (for POST). Participants were told to refrain from physical activity prior to the testing sessions, and participants voided their bladders during urinalysis described below. The following tests were performed during each testing session:

#### Hydration status and profile of mood state

Participants were instructed to submit a urine sample (~5 mL) to assess normal hydration specific gravity levels (1.005–1.020 ppm) using a handheld refractometer (ATAGO; Bellevue, WA, USA). Participants with a urine specific gravity >1.020 were asked to consume 400 ml tap water and were re-tested ~10 min later. Following urinalysis, profiles of mood state (POMS) were collected on Google Forms using the questionnaire published by Grove and Prappavessis (18). Total mood disturbances (TMD) were calculated by summing negative emotion scores and subtracting positive emotion scores from this summed value.

#### Algometry

Following the POMS questionnaire, pressure-to-pain threshold (PPT) of the outer aspect of the right upper thigh was measured using a handheld algometer (Force Ten FDX, Wagner Instruments, Greenwich, CT, USA) according to methods described in our previous work (19). Briefly, focal pressure was applied by the algometer to proximal, medial, and distal portions of the right vastus lateralis (VL) which were marked for accurate application of force. Algometry pressure was applied at a rate of approximately 5 Newtons (N) per second at each site until the participant audibly indicated the specific moment at which the applied pressure became painful. At this point, the PPT value in N was recorded. The digital display of the algometer indicating the force value was blinded to participants. The PPT was measured sequentially at proximal, medial, and distal sites, respectively, three times for triplicate measures with ~30 s between cycles of measurement. The average of the triplicate measures at each site was calculated as the respective PPT of the site, and these values were averaged for a total PPT.

#### Body composition assessment

Following algometry, height and body mass were assessed using a digital column scale (Seca 769; Hanover, MD, USA) with weights and heights being collected to the nearest 0.1 kg and 0.5 cm, respectively. After this, participants partook in a full body dual x-ray absorptiometry (DXA) scan (Lunar Prodigy; GE Corporation, Fairfield, CT, USA). All DXA scans were completed by the same investigator (M.A.R.). According to previous data published by our laboratory (20), the same-day reliability of the DXA during a test-calibrate-retest on 10 participants produced an intra-class correlation coefficient (ICC) of 0.998 for total body lean mass.

#### Ultrasound muscle thickness measurements

Participants also underwent duplicate ultrasound assessments on the right side of the body during each testing session to determine average right leg VL muscle and right bicep brachii thicknesses with a 3–12 MHz multi-frequency linear phase array transducer (Logiq S7 R2 Expert; General Electric, Fairfield, CT, USA). VL measurements were taken at the midway point between the iliac crest and patella of the right femur, which was marked with a cross for probe placement. Participants were instructed to stand and displace bodyweight more to the left leg to ensure the right leg was relaxed. Thereafter, the probe was placed horizontally at the previously marked location and an image was captured. The probe was removed, and the aforementioned steps were repeated for a second subsequent image. Similarly, bicep brachii thickness measurements were taken ~60% distal from the acromial process of the scapula to the lateral epicondyle of the humerus, which was marked with a cross for probe placement. Thereafter, the probe was placed horizontally at the previously marked location and an image was captured. The probe was removed, and the aforementioned steps were repeated for a second subsequent image. All ultrasound assessments were completed by the same investigator (P.W.M.). Reliability of duplicate ultrasound muscle thickness measurements on 33 participants at PRE produced an ICC of 0.994.

#### Total body water assessment

Total body water (TBW), extracellular water (ECW), and intracellular water (ICW) were measured by bioimpedance spectroscopy (BIS) using the SFB7 device (ImpediMed Limited, Queensland, AU) according to the methods described by Moon et al. (21). The SFB7 device measures whole-body bioelectrical impedance at more than 200 frequencies, and uses complex Cole models to estimate TBW, ICW and ECW. Moreover, the SFB7 device: (a) has excellent agreement with TBW assessed via deuterium oxide (21), (b) has excellent agreement with ECW assessed via sodium bromide dilution (22), and (c) has been posited to be the best non-invasive methodology for the determination of fluid compartmentalization (23). This test involved the participant resting in a supine position for 5–10 min, after which TBW, ICW, and ECW estimates were determined while the participants laid supine on a table with his arms ≥30° away from the torso and legs separated. The average of two readings was used to represent the participants' TBW, ICW, and ECW. All BIS tests were supervised by the same investigator (K.C.Y.). Reliability of duplicate TBW measurements on 24 participants at PRE produced an ICC of 0.999. Notably, while unadjusted whole body DXA LBM raw scores are presented herein, we also calculated changes in DXA LBM from weeks 1–3 and weeks 3–6 (ΔLBM) corrected for changes in ECW at these time points (i.e., ECW-corrected ΔLBM). This correction is illustrated in the equation below:

$$\begin{array}{l} ECW-corrected\;\Delta LBM=POST\;\left(or\;MID\right)\;DXA\;LBM\\ \;-PRE\;DXA\;LBM\\ \;-[POST\;\left(or\;MID\right)\;BIS\;ECW\\ \;-PRE\;BIS\;ECW] \end{array}$$

The justification for this correction comes from literature suggesting expansions of ECW being representative of edema or inflammation, and such expansions potentially masking true alterations in functional skeletal muscle mass (24).

#### Muscle biopsies and tissue processing

After body composition and ultrasound measurements, VL muscle biopsies from the right leg were collected using a 5-gauge needle under local anesthesia as previously described (25). Immediately following sampling, tissue was teased of blood and connective tissue, and ~20-40 mg was embedded in cryomolds containing optimal cutting temperature (OCT) media (Tissue-Tek®, Sakura Finetek Inc.; Torrence, CA, USA). Embedding was performed by positioning the tissue in cryomolds for perpendicular slicing in a non-stretched state prior to rapid freezing. Cryomolds were then frozen using liquid nitrogen-cooled isopentane and subsequently stored at −80°C until immunofluorescent staining for determination of fiber cross sectional area (fCSA). The remaining tissue was wrapped in pre-labeled foils, flash frozen in liquid nitrogen, and subsequently stored at −80°C. All biopsies were obtained by the same investigators (M.D.R. and C.T.H.), and biopsies were obtained ~2 cm apart at the same approximate depth each testing session.

#### Immunohistochemistry for fiber cross sectional area assessment

Similar methods for immunohistochemistry have been employed previously in our laboratory (25). Sections from OCT-preserved samples were cut at a thickness of 8 μm using a cryotome (Leica Biosystems; Buffalo Grove, IL, USA) and were adhered to positively-charged histology slides. Once all samples were sectioned, batch processing occurred for immunohistochemistry. During batch processing sections were air-dried at room temperature for 10 min, permeabilized in a phosphate-buffered saline (PBS) solution containing 0.5% Triton X-100 for 10 min, and blocked with 100% Pierce Super Blocker (Thermo Fisher Scientific) for 10 min. For fiber type staining, sections were subsequently washed for 2 min in PBS. Sections were then incubated for 10 min with a pre-diluted commercially-available rabbit anti-dystrophin IgG antibody solution (catalog #: GTX15277; Genetex Inc.; Irvine, CA, USA) and spiked in mouse anti-myosin I IgG (catalog #: A4.951 supernatant; Hybridoma Bank, Iowa City, IA, USA; 40 μL added per 1 mL of dystrophin antibody solution). Sections were then washed for 2 min in PBS and incubated in the dark for 15 min with a secondary antibody solution containing Texas Red-conjugated anti-rabbit IgG (catalog #: TI-1000; Vector Laboratories, Burlingame, CA, USA), and Alexa Fluor 488-conjugated anti-mouse IgG (catalog #: A-11001; Thermo Fisher Scientific) (~6.6 μL of all secondary antibodies per 1 mL of blocking solution). Sections were washed for 2 min in PBS, air-dried, and mounted with fluorescent media containing 4,6-diamidino- 2-phenylindole (DAPI; catalog #: GTX16206; Genetex Inc.). Following mounting, slides were stored in the dark at 4°C until immunofluorescent images were obtained. After staining was performed on all sections, digital 10x objective images were captured using a fluorescence microscope (Nikon Instruments, Melville, NY, USA). All images were captured by a laboratory technician who was blinded to the group assignment of each participant. Approximate exposure times were 400 ms for TRITC and FITC imaging. Our staining method allowed the identification of cell membranes (detected by the Texas Red filter), type I fiber green cell bodies (detected by the FITC filter), type II fiber black cell bodies (unlabeled), and myonuclei (detected by the DAPI filter). Measurements of type I and II fCSAs were performed using custom-written pipelines in the open-sourced software CellProfiler^TM^ (26) per modified methods previously described whereby the number of pixels counted within the border of each muscle fiber was converted to a total area (μm^2^). A calibrator slide containing a 250,000 μm^2^ square image was also captured, and pixels per fiber from imaged sections were converted to area using this calibrator image. On average, 113 ± 26 fibers per cross-section were identified for analysis at each sampling time. A *post-hoc* experiment performed in our laboratory to examine potential differences in fCSA measurements between sections on the same slide (*n* = 23 slides) revealed strong reliability using this method (ICC = 0.929).

### Statistical analysis

Statistical tests were performed in RStudio (Version 1.0.143; R Foundation for Statistical Computing, Vienna, AT), SPSS (Version 23; IBM SPSS Statistics Software, Chicago, IL, USA), and Google Sheets. Group [3 levels (WP, GWP, MALTO)] and time [3 levels (PRE, MID, POST), or 6 levels (Week 1–6) for weekly measures] served as independent variables. A mean-centered covariate for each baseline measurement was added as a parameter to models to examine the explained variance in dependent variables relative to values at PRE. Since nutrition-related data were not available at PRE, and only after collection of data during week 1, no covariate was utilized in this model and a repeated-measures ANOVA was performed after assumptions testing. Statistical assumptions tests were completed prior to analysis consisting of: (1) Shapiro-Wilks tests of residual distributions for normality, (2) Levene's test of homogeneity of variance, and (3) Mauchly's test for Sphericity, given that a repeated-measures analysis of covariance (ANCOVA) was performed for the provision of *p*-values. Violation of these assumptions and appropriate data transformations (i.e., square root or log_10_ transformations) when residuals were not normally distributed were completed prior to ANCOVA for the avoidance of type 1 or type 2 errors. Data transformation and data removal were avoided with intention to analyze all raw data. For this reason, if the majority of levels of group (2 of 3 groups) at each level of time were normally distributed, ANCOVA proceeded without data transformation. If the assumptions of homogeneity of variance or sphericity were violated, Greenhouse-Geisser corrections to degrees of freedom were made. The alpha level of significance was set at *p* < 0.05. For significant main effects of time and group × time interactions, LSD *post-hoc* tests were performed at each level of time to elucidate specific differences. A priori power analysis in RStudio using general linear model parameters in the “pwr” package (Version 1.2-1) revealed 84.5% power (power = 1 – β) for the discovery of a large effect size when 2 predictors and 31 observations were employed [e.g., *k* = 2 (time, y-intercept), *n* = 31 (31 participants), *f* ^2^ = 0.35 (large effect), *p* = 0.05 (a-priori level of significance)]. However, a power analysis to detect a significantly large difference of an effect between groups when 3 groups (*k* = 3) included 10 participants each (*n* = 10) revealed 44% power. Therefore, Cohen's d effect sizes and 95% confidence intervals were also calculated for each dependent variable, aside from nutrition data, to examine mean differences between groups from PRE to POST considering the pooled standard deviation of a dependent variable at baseline since population-based inferences were underpowered. Supplementary Tables 2–11 provide descriptive statistics, effect sizes, and 95% confidence intervals for each dependent variable. Additionally, raw data are provided in .csv files (Supplementary SDC 1, Supplementary SDC 2) and supplementary tables including effect size calculations and 95% confidence intervals are provided in a .pdf file (Supplementary SDC 3).

## Results

### Self-reported nutrition

Nutritional analyses were performed on participants who logged >90% of days throughout the study. Twelve participants irregularly reported or did not report nutritional intakes each week resulting in 19 complete sets of nutritional data. Hence, Table 2 contains self-reported dietary intakes from these 19 participants. For these participants, no significant main effect of group, time, or group × time interaction was observed for self-reported absolute or relative energy, protein, or carbohydrate intakes (*p* > 0.05). A significant main effect of time and group, but no interaction, was observed on reported fat intake. Fat intake decreased over time (*p* = 0.006), and WP averaged higher reported intakes than GWP and MALTO (*p* = 0.017). In reference to participant adherence to nutrition recommendations provided by the R.D., GWP reported less protein consumption than recommended during weeks 1 and 6 (*p* < 0.05), and the reported consumption of dietary fat relative to that recommended was significantly different during weeks 1–6 in MALTO, weeks 1–3 in GWP, and weeks 4–6 in WP (*p* < 0.05).

**Table 2:** Self-reported dietary data.

	**week**	**MALTO (*****n*** = **6)**				**WP (*****n*** = **6)**				**GWP (*****n*** = **7)**				**TOTAL (*****n*** = **19)**			
		**Abs**	**SD**	**Rel**	**SD**	**Abs**	**SD**	**Rel**	**SD**	**Abs**	**SD**	**Rel**	**SD**	**Abs**	**SD**	**Rel**	**SD**
Energy (kcal/d) or (kcal/kg/d)	1	2,870	462	35.2	5.4	2994	462	35.2	5.4	2,625	845	32.2	10.3	2819	595	34.1	7.0
	2	2,733	583	36.0	6.9	3065	583	36.0	6.9	2,832	582	34.7	7.1	2874	520	34.7	6.1
	3	2,737	614	34.9	7.3	2959	614	34.9	7.3	2,677	515	32.6	6.3	2785	479	33.5	5.7
	4	2,827	211	38.6	2.4	3288	211	38.6	2.4	2,744	573	33.3	6.9	2942	442	35.2	5.2
	5	2,912	281	37.5	3.2	3199	281	37.5	3.2	2,558	827	30.9	10.1	2872	586	34.3	7.0
	6	2,209	350	35.8	4.2	3047	350	35.8	4.2	2,319	903	28.0	10.8	2514	828	30.1	10.0
PRO (g/d) or (g/kg/d)	1	148	38	2.1	0.5	178	38	2.1	0.5	186	61	2.3	0.8	171	45	2.1	0.4
	2	151	27	2.1	0.2	181	27	2.1	0.2	184	48	2.3	0.5	173	36	2.1	0.4
	3	166	52	2.3	0.7	191	52	2.3	0.7	170	53	2.1	0.5	176	46	2.1	0.4
	4	183	38	2.4	0.5	205	38	2.4	0.5	187	56	2.3	0.8	191	44	2.3	0.4
	5	190	57	2.5	0.7	214	57	2.5	0.7	184	82	2.2	1.1	195	65	2.3	0.9
	6	172	55	2.4	0.7	204	55	2.4	0.7	168	87	2.0	1.1	181	79	2.2	0.9
CHO (g/d) or (g/kg/d)	1	278	91	3.1	1.0	261	91	3.1	1.0	251	87	3.1	1.1	263	78	3.2	0.9
	2	260	53	3.1	0.7	262	53	3.1	0.7	279	55	3.4	0.8	268	50	3.2	0.4
	3	254	45	3.1	0.5	267	45	3.1	0.5	267	47	3.3	0.5	263	47	3.2	0.4
	4	271	41	3.4	0.5	286	41	3.4	0.5	263	76	3.2	0.8	273	53	3.3	0.4
	5	288	28	3.5	0.2	298	28	3.5	0.2	251	79	3.0	1.1	277	67	3.3	0.9
	6	207	35	3.4	0.5	290	35	3.4	0.5	211	91	2.5	1.1	234	82	2.8	0.9
FAT (g/d) or (g/kg/d)	1	123	21	1.7	0.2	141	21	1.7	0.2	102	39	1.3	0.5	121	33	1.5	0.4
	2	118	27	1.6	0.2	137	27	1.6	0.2	110	28	1.3	0.3	121	27	1.5	0.4
	3	111	33	1.5	0.5	130	33	1.5	0.5	101	26	1.2	0.3	113	27	1.4	0.4
	4	113	12	1.7	0.2	146	12	1.7	0.2	107	29	1.3	0.3	121	27	1.4	0.4
	5	108	15	1.5	0.2	126.7	15	1.5	0.2	91	32	1.1	0.3	108	25	1.3	0.4
	6	82	20	1.5	0.2	129.7	20	1.5	0.2	90	31	1.1	0.3	100	33	1.2	0.4

*All data absolute (Abs) or relative (Rel) self-reported dietary intake data are presented as means ± standard deviation (SD) values. Data from only 19 participants were included in the nutritional analyses given that 12 participants irregularly reported (or did not report) nutritional intakes. No significant main effect of group, time, or group × time interaction was observed for reported absolute or relative calories, protein intake, or carbohydrate intake (p > 0.05); thus no significance is indicated*.

### Training volume, soreness, BB velocity, and total mood disturbance

**Figure 2:**
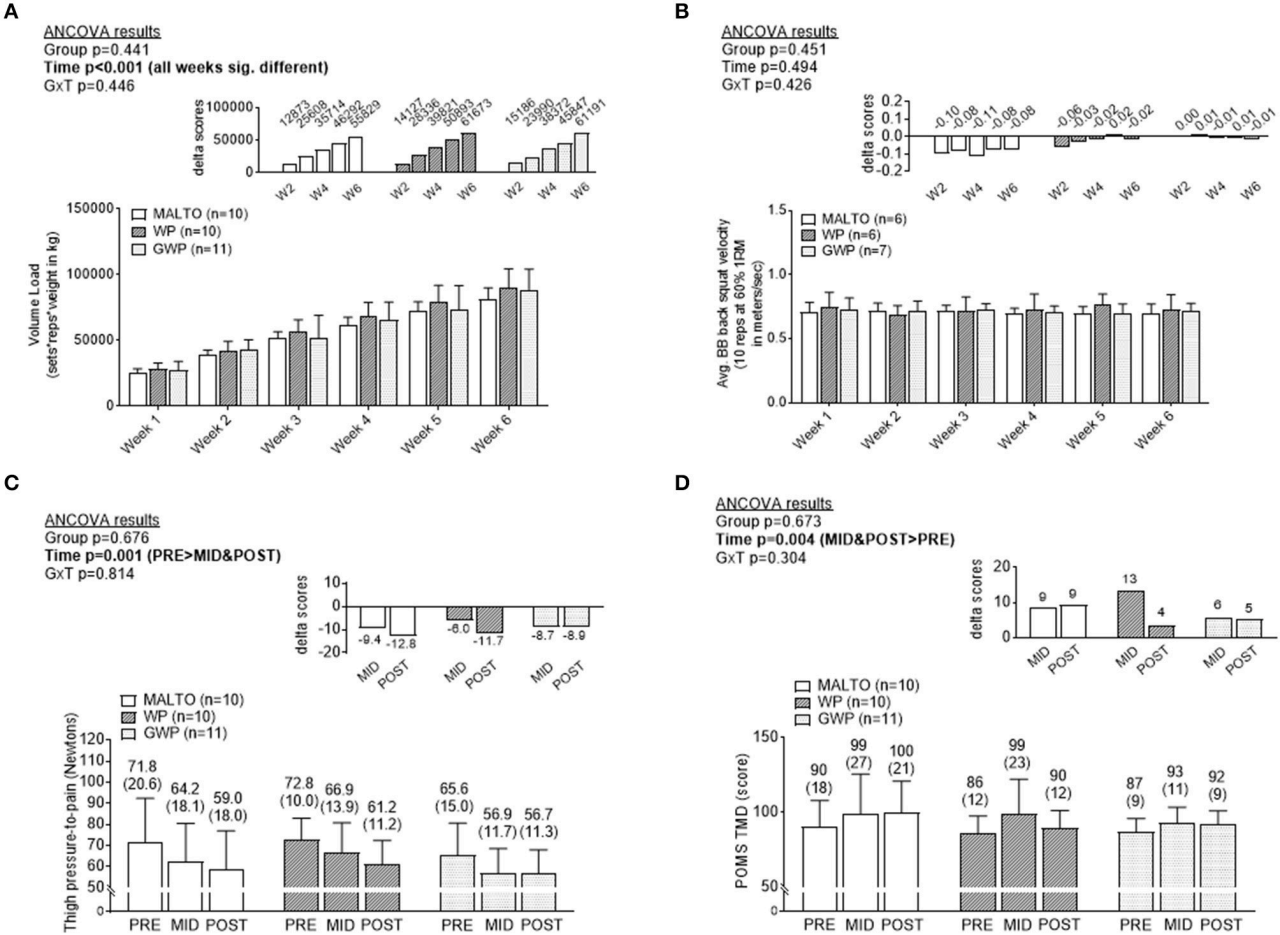
Differences in training volume, back squat lifting velocity, thigh soreness, and total mood disturbance among supplementation groups. Only a significant time effect was observed for training volume with values increasing on a weekly basis **(A)**. No main effects or group × interaction was observed for back squat lifting velocity **(B)**. Only a significant time effect was observed for thigh pressure-to-pain values (lower values indicates greater soreness) **(C)**. Only a significant time effect was observed for profile of mood state (POMS) total mood disturbance (TMD) (greater values indicates more mood disturbance) **(D)**. All data are presented as means ± standard deviation values, and values in **(C,D)** are indicated above each bar; values for panels a and b are not indicated due to space constraints but are provided in the raw data file. Additionally, each data panel has delta values from PRE included as inset data. MALTO, maltodextrin group; WP, standardized whey protein group; GWP, graded whey protein group.

Training volume significantly increased over time on a weekly basis (*p* < 0.001), but no significant group or group × time interaction was observed (Figure 2A, Supplementary Tables 13, 14). No significant main effects or group × time interaction was observed for BB velocity assessed during set 1 of the back squat exercise at the beginning of each Friday training session (Figure 2B). Algometry PPT measures significantly decreased over time (*p* < 0.001), but no significant group or group × time interaction was observed (Figure 2C). PPT was significantly lower at MID compared to PRE (*p* = 0.002), and POST compared to PRE (*p* < 0.001), but not at POST compared to MID (*p* = 0.122). POMS TMD significantly increased over time (*p* = 0.002) but no significant effect of group or group × time interaction was observed (Figure 2D). TMD was significantly higher at MID compared to PRE (*p* = 0.002), and at POST compared to PRE (*p* < 0.001), but not at POST compared to MID (*p* = 0.254).

### Body composition data

**Figure 3:**
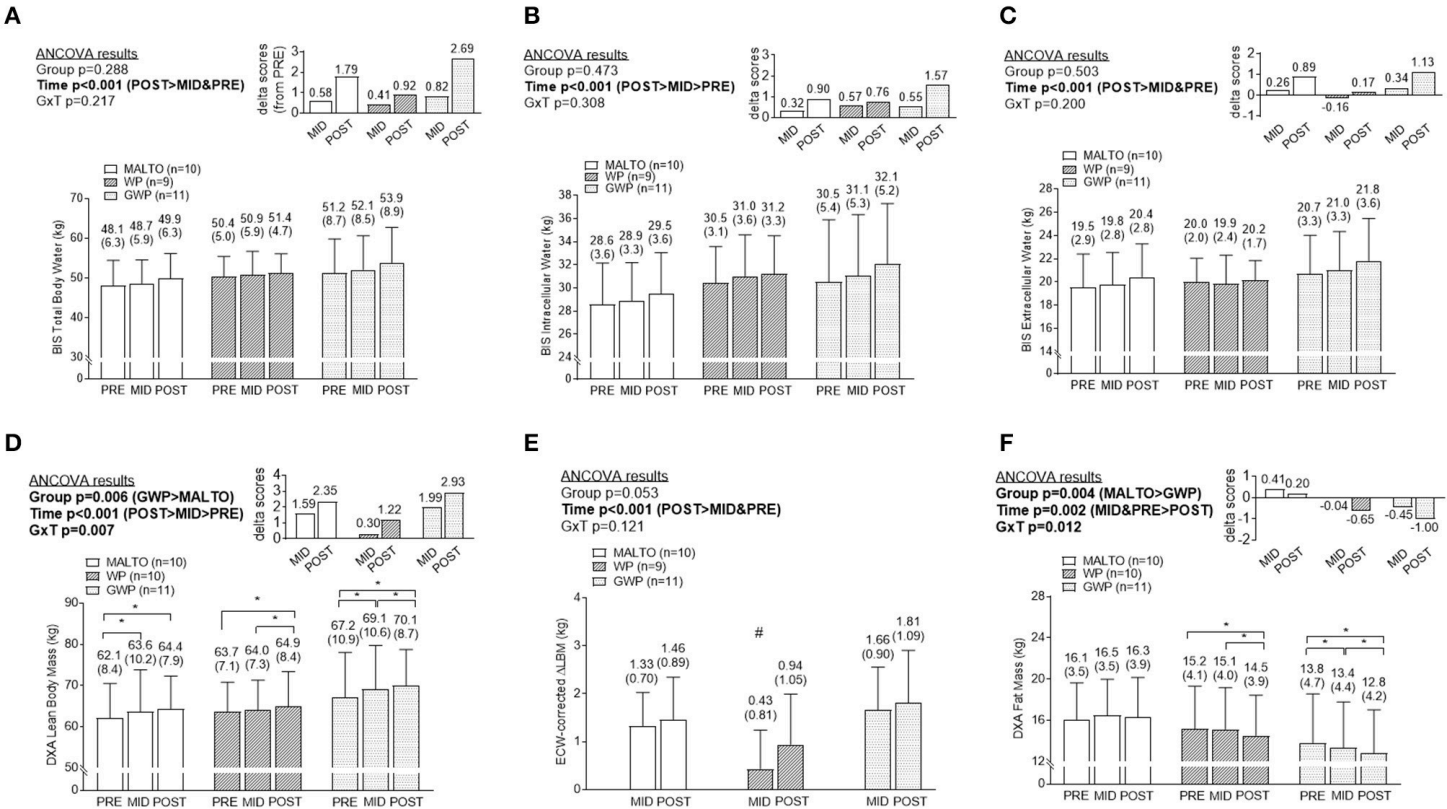
Body composition differences between supplementation groups. Only significant time effects were observed for total body water content **(A)** assessed via bioelectrical impedance spectroscopy (BIS), BIS intracellular water content **(B)**, and BIS extracellular water content **(C)**. For all of these metrics, POST values were significantly greater than PRE and MID values. Significant main group and time effects as well as a group × time interaction were observed for lean body mass **(D)** assessed via dual x-ray absorptiometry (DXA). *Post-hoc* tests indicated lean body mass increased within groups from PRE to MID (MALTO and GWP; *p < 0.05), MID to POST (WP abd GWP; *p < 0.05), and PRE to POST (all groups; *p < 0.05). However, no significant between-group differences existed at each level of time. A significant main time effect as well as a group × time interaction was observed for change scores in DXA lean body mass corrected for change scores in ECW **(E)**. *Post-hoc* tests indicated this metric increased within groups from PRE to MID (MALTO and GWP; *p < 0.05), and PRE to POST (all groups; *p < 0.05). Additionally, MID WP was significantly lower than MID GWP (*#p* = 0.004). Significant main group and time effects as well as a group × time interaction were observed for fat mass **(F)** assessed via DXA. *Post-hoc* tests indicated fat mass decreased within groups from PRE to MID (GWP; *p < 0.05), MID to POST (WP and GWP; *p < 0.05), and PRE to POST (WP and GWP; *p < 0.05). However, no significant between-group differences existed at any level of time. All data are presented as means ± standard deviation values, and values are indicated above each bar. Additionally, each data panel (except **E**) has delta values from PRE included as inset data. MALTO, maltodextrin group; WP, standardized whey protein group; GWP, graded whey protein group.

TBW significantly increased over time (*p* < 0.001), but no significant group or group × time interaction was observed (Figure 3A). Both ICW (Figure 3B) and ECW (Figure 3C) significantly increased over time, but no significant group or group × time interactions were observed for these metrics.

DXA LBM significantly increased over time (*p* < 0.001; Figure 3D). A significant group × time interaction (*p* = 0.007) was observed for LBM, although LSD *post-hoc* tests revealed no significant differences among groups at any sampling time. When corrected for changes in ECW, a significant increase in DXA LBM was observed from PRE to POST (*p* < 0.001). No significant group or group × time interaction was observed (Figure 3E). DXA fat mass significantly decreased over time (*p* = 0.004; Figure 3F). A significant group × time interaction (*p* = 0.012) was observed and, while LSD *post-hoc* tests revealed no significant differences among groups at any sampling time, the difference between GWP and MALTO at MID and POST approached significance (*p* = 0.088 and *p* = 0.064, respectively).

### Segmental DXA data

**Figure 4:**
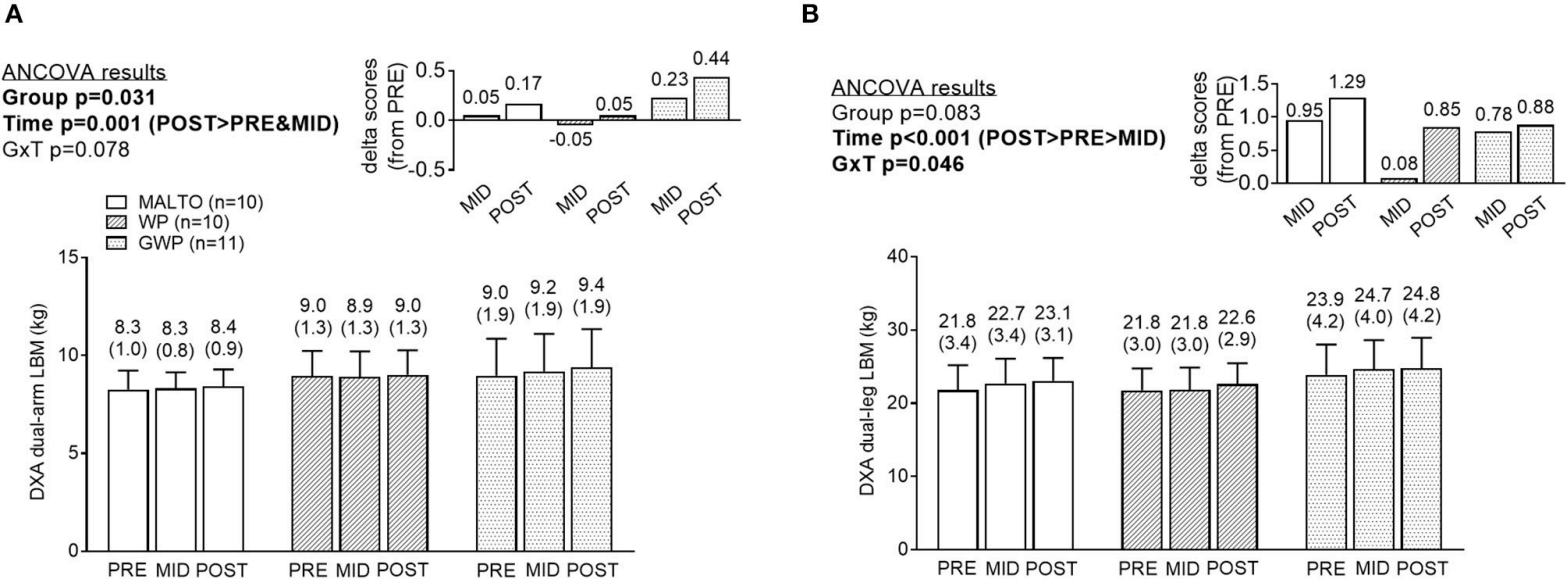
Segmental DXA data differences between supplementation groups. Significant time effects were observed for DXA dual-arm LBM **(A)** and DXA dual-leg LBM **(B)**, with MID, and/or POST values being greater than PRE. While a significant group × time interaction was observed for DXA dual-leg LBM, no significant between-group differences at each time point were observed. All data are presented as means ± standard deviation values, and values are indicated above each bar. Additionally, each data panel has delta values from PRE included as inset data. MALTO, maltodextrin group; WP, standardized whey protein group; GWP, graded whey protein group.

DXA dual-arm LBM significantly increased over time (*p* = 0.001; Figure 4A). Additionally, DXA dual-leg LBM significantly increased over time (*p* < 0.001; Figure 4B), and there was a significant group × time interaction (*p* = 0.046). However, LSD *post-hoc* tests revealed no significant differences among groups at any sampling time.

### Muscle thicknesses and fCSA

**Figure 5:**
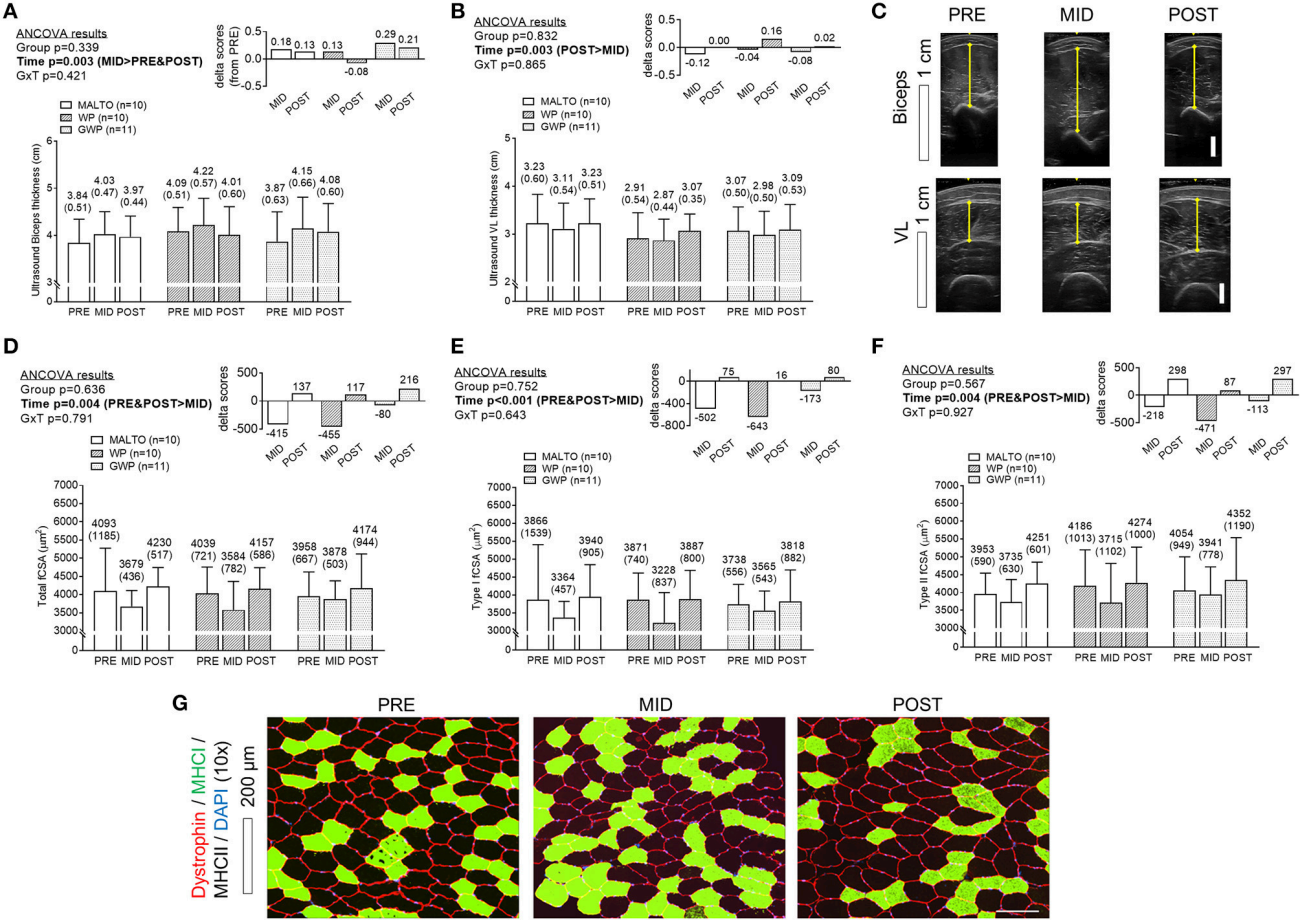
Muscle thickness and VL fiber size differences between supplementation groups. Only a significant time effect was observed for biceps thickness **(A)** assessed via ultrasound with MID values being greater than PRE- and POST values. Only a significant time effect was observed for VL thickness **(B)** assessed via ultrasound with MID values being less than POST values. Panel **(C)** provides representative images of ultrasound scans from the same participants. Only significant time effects were observed for total fiber cross sectional area (fCSA) **(D)**, type I fCSA **(E)**, and type II fCSA **(F)** assessed via histology with MID values being less than PRE and POST values. Panel **(G)** provides representative 10x objective histology images from VL biopsies of the same participant. All data are presented as means ± standard deviation values, and values are indicated above each bar. Additionally, each data panel has delta values from PRE included as inset data. MALTO, maltodextrin group; WP, standardized whey protein group; GWP, graded whey protein group.

A significant effect of time was observed for bicep thickness with a greater thickness at MID compared to PRE (*p* = 0.001) and POST (*p* = 0.040), but there was no significant group × time interaction (Figure 5A). A significant effect of time was also observed for VL thickness (*p* = 0.003) with *post-hoc* tests revealing lower values at MID compared to POST (*p* < 0.001), and lower values at MID compared to PRE approaching significance (*p* = 0.053; significance Figures 5B,C). However, a significant group × time interaction was not observed. When summing biceps and VL thicknesses at each level of time, there were no significant differences between groups at each level of time. However, a significant main effect of time revealed that the summed values of thickness measurements were significantly higher at POST compared to PRE (*p* = 0.049). The summed value at POST was 7.16 ± 0.77 cm where the summed value was 6.98 ± 0.81 cm at PRE (data not shown). Significant reductions in VL total fCSA, type I fCSA, and type II fCSA were observed from PRE to MID (*p* = 0.045, *p* = 0.009, and *p* = 0.0410, respectively), followed by a significant increase from MID to POST (*p* = 0.004, *p* = 0.004, and *p* = 0.001, respectively) (Figures 5D–F, Supplementary Table 12). However, values in these metrics at POST were not significantly different from values at PRE, and no significant group or group × time interactions were observed.

### Training volume vs. change in DXA lean body mass

**Figure 6:**
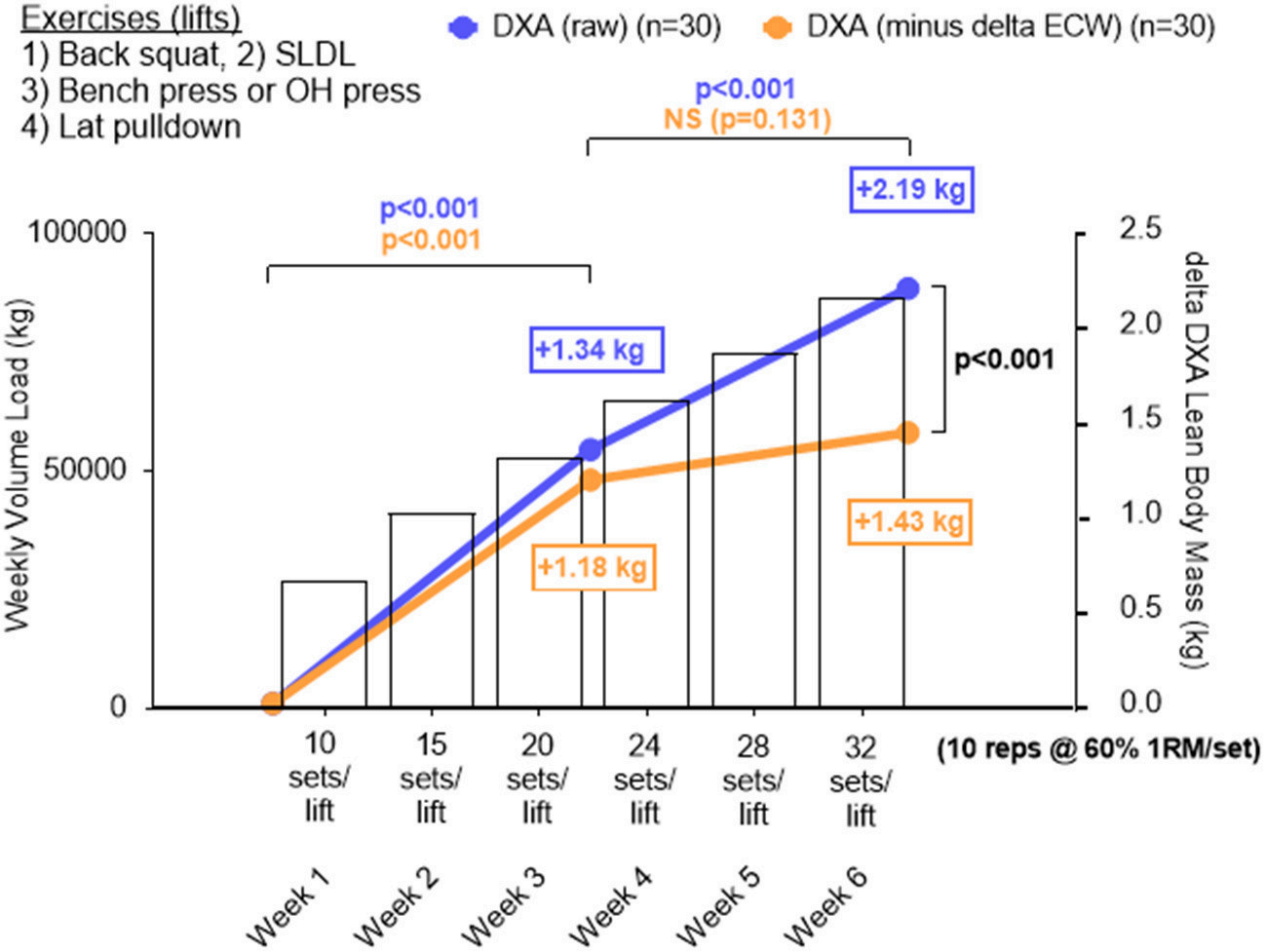
Change in DXA lean body mass plotted against increases in training volume for all participants. Data in this figure include DXA lean body mass changes (blue line graph), changes in LBM by subtracting changes in extracellular water (i.e., ECW-corrected ΔLBM), and training volume (bar data) from all 30 participants who underwent DXA and BIS testing. A significant increase in LBM from PRE to MID (*p* < 0.001) and MID to POST (*p* < 0.001) was observed in DXA LBM and this was proportional to the increase in training volume over time. When considering ECW-corrected ΔLBM changes, a similar increase occurred across groups from PRE to MID (*p* < 0.001), but the increase from MID to POST was not significant (*p* = 0.131). Additionally, POST DXA LBM was significantly higher than POST ECW-corrected ΔLBM. All data is presented as mean changes, and bars depicting standard deviation were left off of these panels in order to simplify the figure.

As previously stated, a primary goal was to determine the whole-body hypertrophic response in all participants given that the RT volume is the highest ever attempted in a laboratory-based study over a 6-week period. Interestingly, a significant increase in LBM occurred from PRE to MID (*p* < 0.001) and MID to POST (*p* < 0.001), and these increases were proportional to the significant increase in weekly training volume (Figure 6). When considering ECW-corrected ΔLBM, a similar increase was observed from PRE to MID (*p* < 0.001), but a non-significant increase from MID to POST (*p* = 0.131).

## Discussion

Large numerical increases in DXA LBM (+2.93 kg on average) and significant reductions in DXA fat mass (−1.00 kg on average) were observed in the GWP group. However, it is also notable that the MALTO group experienced a PRE to POST increase DXA LBM (+2.35 kg, *p* < 0.05), and the GWP and MALTO groups experienced similar PRE to POST increases in type II muscle fiber cross-sectional area (+~300 μm^2^). Thus, similar hypertrophic effects observed in the MALTO group preclude us from suggesting that GWP supplementation is clearly superior to MALTO supplementation in facilitating skeletal muscle hypertrophy.

As stated prior, several studies have indicated that single dose ingestion or longer-term supplementation with higher whey protein doses enhances anabolic outcomes. For example, Macnaughton et al. (4) recently reported significantly greater MPS responses to a resistance exercise bout and whey protein ingestion when 40 g were consumed post-exercise compared to 20 g. Additionally, Witard et al. (3) compared myofibrillar protein synthesis responses following the ingestion of 40 g of whey protein to 0, 10, and 20 g in younger resistance-trained males. These authors noted numerically larger, but not significantly different, responses from ingestion of 40 vs. 20 g, while 0 and 10 g resulted in significantly lower responses. Regarding longer-term supplementation data, and as stated previously, four studies in previously-trained subjects have reported that high-dose (80–120 g/d) supplementation with whey protein (or a protein blend containing whey protein) significantly increases LBM following 6–12 weeks of RT (9–12). Additionally, Antonio et al. (27) reported ~2 kg increases in LBM (assessed via air displacement plethysmography) in a group of 20 participants consuming ~4.4 g/kg/day of dietary protein over an 8-week period, much of which was supplemented via whey protein in the diet, compared to ~1.3 kg increases in LBM in another group of participants consuming ~1.8 g/kg/day. Antonio et al. (28) conducted a follow-up investigation wherein a total of 31 participants consumed ≥3 g/kg/d, and 17 participants consumed their normal amount of dietary protein (1.8–2.3 g/kg/d) for 8 weeks while undergoing 5 days of RT per week. These authors reported statistically equivalent LBM gains in both groups (+1.5 kg), although the three highest hypertrophic responders in the study consumed ≥3 g/kg/d. Collectively, our data and these previous reports suggest that high daily whey protein intake appears to promote skeletal muscle hypertrophy. However, given that MALTO supplementation herein also promoted similar anabolic effects and subjects from all groups self-reported consuming >2.0 g/kg/d of dietary protein, it does not appear that high-dose whey protein supplementation during high volume RT in lieu of adequate protein intake (i.e., >1.6 g/kg/d) is clearly superior in promoting hypertrophy.

Our data suggesting GWP promotes the greatest loss in fat mass is intriguing and agrees with prior literature. For instance, Cribb et al. (10) reported that participants supplementing with ~120 g/d of whey protein lost a significant amount of fat mass compared to casein-supplemented participants (−1.4 vs. +0.1 kg). Additionally, Antonio et al. (28) reported that participants consuming high amounts of protein lost significantly more fat mass relative to a lower protein intake group (−1.6 vs. −0.3 kg). There are mechanistic rodent data (29, 30) and longer-term human data (13) suggesting whey protein possesses lipolytic properties. However, this research has mainly indicated that hydrolyzed whey protein could possess lipolytic properties rather than whey protein concentrate; the latter being the source of whey provided to the WP and GWP groups. Given that self-reported caloric intakes were non-significantly but numerically lower on a weekly basis in GWP vs. WP and MALTO participants throughout the study, an alternative explanation of our data could be that the observed loss in fat mass in the GWP group occurred due to a lower calorie intake relative to the other groups. Indeed, these data agree with studies which have mechanistically demonstrated that whey protein consumption acutely increases circulating levels of satiety-related hormones and reduces food intake [reviewed in (31)].

Beyond the observed supplementation effects, a unique finding of this investigation is the apparent dose-response relationship observed between RT volume and LBM changes corrected for alterations in ECW (Figure 6). It has been suggested that a positive relationship exists between RT volume and skeletal muscle hypertrophy up to a certain volume threshold (32). A recent meta-analysis by Schoenfeld et al. (14) demonstrated significantly greater hypertrophic responses after completion of 10 sets per week of a resistance exercise emphasizing specific musculature compared to <5 sets per week. However, others have suggested that a plateau in the hypertrophic response exists beyond select RT doses (33). Our data indicate no clear plateau in RT-induced muscle mass increases when RT volumes are increased from 10 sets of 10 repetitions at 60% 1RM per exercise per week up to 32 sets per week, and this interpretation stems from the significant increases observed in DXA LBM from weeks 1 to 3 and 3 to 6. However, when changes in DXA LBM were corrected for changes in ECW, a different interpretation arises. Notably, subtractions of ECW changes from LBM changes were completed in an attempt to control for transient extracellular fluid retention (e.g., local swelling) related to tissue trauma potentially due to the extreme RT volumes completed by participants. In this regard, Yamada et al. (24) suggest expansions of ECW may be representative of edema or inflammation and can mask true alterations in functional skeletal muscle mass. Further, these authors suggest the measurements of fluid compartmentalization (e.g., ICW, ECW), which are not measured by DXA, are needed if accurate representation of functional changes in LBM are to be inferred. When ECW-corrected ΔLBM changes are considered, week 1–3 increases were similar in magnitude to uncorrected DXA LBM changes (+1.18 vs. +1.34 kg, respectively). However, ECW-corrected ΔLBM changes from weeks 3–6 were significantly lower than uncorrected DXA LBM changes (+0.85 vs. +0.25 kg, respectively). We speculate the latter observation could be related to local inflammation or edema induced by increasing RT volume above 20 sets per exercise per week. Thus, when considering uncorrected DXA LBM changes, one interpretation of these data is that participants did not experience a hypertrophy threshold to increasing volumes up to 32 sets per week. However, if accounting for ECW changes during RT does indeed better reflect changes in functional muscle mass, then it is apparent participants were approaching a maximal adaptable volume at ~20 sets per exercise per week. First, it is critical to note that more research is needed in order to determine if correcting changes in DXA LBM relative to changes in ECW is a valid method which better reflects changes in functional muscle mass. Second, and in regard to a set volume threshold, we are careful to generalize these findings across populations to avoid promotion of an assumed RT volume ceiling for eliciting hypertrophy since there is likely no “one size fits all” RT dose for eliciting a maximal hypertrophic response (34, 35). Rather, optimally dosing RT for hypertrophic outcomes should depend on the physiological status of an individual and particularly as it pertains to recent historical training (36).

Other interesting effects related to training emerged from the current study. First, divergent adaptive responses in the bicep brachii and VL muscles as assessed via ultrasound were observed. Specifically, increases in biceps thickness and decreases in VL thickness occurred from PRE to MID and the inverse effects occurred from MID to POST. While fiber type data in human biceps brachii muscle are lacking, Dahmane et al. (37) reported ~60% of fibers in the biceps brachii were type II, while ~40% were type I. Herein, we observed the VL consisted of ~50% type II fibers, on average. Type II fibers typically hypertrophy to a greater extent in response to RT relative to type I fibers (38), and the observed divergent responses in the biceps and VL muscle thickness measurements may be related to fiber-type distributions of these muscles. However, this hypothesis is speculative at best and more work is needed to determine how different muscle groups mechanistically adapt to high volume RT. Another striking observation was the PRE to MID decrease in VL thickness and fCSA values followed by the MID to POST increase in these metrics. Damas et al. (36) recently reported significant increases in muscle damage after a single bout of RT, followed by an attenuation of damage measured from a similar bout 3 and 10 weeks later. Additionally, while these authors observed significant elevations in MPS after bouts at weeks 3 and 10, significant increases in fCSA were only observed after 10 weeks. The authors posited that significant increases in muscle damage and MPB from weeks 1–3 outpaced increases in MPS resulting in no significant increase in fCSA until the RT-induced damage response subsided from weeks 3–10. Relating these findings to our data, the initial atrophic VL muscle response during the first 3 weeks of training may have been due to high levels of muscle damage/MPB counteracting increases in MPS. However, during weeks 3–6, MPS levels may have outpaced muscle damage/MPB leading to increases in muscle thickness and fCSA. Again, these findings are speculative at best since we did not assess markers of muscle protein turnover.

### Experimental considerations

Our study is limited in that only 31 participants completed the intervention. As such, we were underpowered to detect small, but significant, effects. Second, an unresolved limitation is that not all participants adhered to the dietary self-reporting protocol. We felt that 2 to 4-day food logs would not entirely reflect what participants consumed throughout the study. For this reason, we sought to implement a convenient and more ecologically valid method of self-reporting dietary data which persuaded our utilization of daily mobile application entries. However, despite consistent verbal encouragement by research staff, only ~60% of participants were adherent. In regard to dietary adherence it is also worth noting that, while our intent was to grade dietary protein intakes on a weekly basis in the WP and GWP groups, all groups (including MALTO) self-reported consuming similar amounts of protein throughout the study (>2.0 g/kg/d). Thus, results observed in the GWP group could be interpreted as physiological effects due to the replacement of dietary protein with whey protein on a weekly basis rather than increases in overall protein intake. We do not propose that this protein replacement strategy should be adopted by recreational lifters or athletes, and future studies should try to resolve if strictly maintaining dietary habits while increasing whey protein dosing promotes physiological benefits. A methodological consideration is our reliance upon DXA assessments reflecting true whole-body muscle mass changes. While numerous forms of body composition assessment exist, the scientific literature supports the utilization of DXA for detecting changes in body composition. Buckinx et al. (39) recently posited DXA as a reference standard (but not gold standard) method for measurement of LBM in research and clinical practice. As mentioned previously, our laboratory has observed excellent same-day reliability of the DXA during a test-calibrate-retest. Notwithstanding, others have suggested a modest overestimation of fat mass using DXA compared to a 4-compartment model of body composition (40). Therefore, we acknowledge that LBM or fat mass assessed via DXA could have been under- or overestimated in an absolute sense. Additionally, PRE to POST increases in DXA dual-arm and DXA dual-leg LBM seemingly did not agree well with the ultrasound data suggesting PRE to POST increases in biceps thickness occurred and only a MID to POST increase in VL thickness occurred. While this finding is difficult to reconcile, it is notable that Franchi et al. (41) have recently reported that change scores in VL ultrasound thickness and VL muscle area assessed via magnetic resonance spectroscopy poorly agree following weeks of RT. Hence, the lack of agreement between DXA and ultrasound could be similarly reflective of between-method comparison limitations reported by Franchi et al. Finally, while a 6-week RT program seems rather abbreviated, we chose to implement this duration due to the concern *a priori* that the implemented volume would lead to injuries past 6 weeks of training. Furthermore, traditional training periodization strategies commonly employed in practical settings organize training phases or “blocks” emphasizing specific adaptations (e.g., hypertrophy, strength) into 3–6 week durations (42). In spite of these limitations, we posit that our findings are novel in the sense that these were the highest RT volumes formally studied in humans to date in a 6-week timeframe.

## Conclusions

GWP participants exhibited robust increases in DXA LBM, and reductions in DXA fat mass. These data imply graded whey protein consumption in conjunction with increases in RT volume (i.e., a proportional supplemental protein hypothesis) is a viable strategy to improve body composition during high volume RT. However, similar PRE to POST effects regarding DXA LBM and VL fCSA changes were observed in the MALTO group and, given that all groups herein consumed >2.0 g/kg/d of dietary protein, this finding suggests that GWP may not provide substantial benefit in promoting hypertrophy when protein intakes are >1.6 g/kg/d as suggested by Morton et al. (5). Additionally, the RT volumes investigated in this study are the highest formally studied in human participants in a 6-week timeframe. Significant increases in LBM corrected for alterations in ECW were observed from weeks 1–3, although this response was dampened from weeks 3–6 suggesting that ~20 sets per exercise per week may approach a maximal adaptable volume in younger resistance-trained men.

## Author contributions

CH was primarily responsible for the design, execution, analysis, and writing of the manuscript. CV critically assisted with all aspects of execution and analysis. All other co-authors assisted in multiple aspects of data collection as well as the preparation of the manuscript. MDR is the principal investigator of the laboratory where much of the work for this study was performed, and assisted in all aspects of the study as well as in the preparation of the manuscript.

### Conflict of interest statement

The results of the study are presented clearly, honestly, and without fabrication, falsification, or inappropriate data manipulation. JM is the Executive Director of Research and Education of Impedimed Inc. who has extensive expertise in body composition assessment. He provided critical considerations for body composition assessments and data interpretation, but his involvement did not influence study results. MI is the Head Science Consultant for Renaissance Periodization who was critically involved in study design and data interpretation.

The remaining authors declare that the research was conducted in the absence of any commercial or financial relationships that could be construed as a potential conflict of interest.

## Supplementary material

Supplementary SDC 1 haun_supplementary_data.csv—this file includes all raw data.

Supplementary SDC 2 haun_supplementary_diet.csv—this file includes dietary recommendations as well as self-reported dietary intakes.

Supplementary SDC 3 haun_supplementary_tables.pdf—this file includes descriptive tables for each dependent variable.

Supplementary Table 1 Pre-study body composition and strength descriptive measurements.

Supplementary Table 2 Body mass.

Supplementary Table 3 Total lean body mass (DXA).

Supplementary Table 4 Total body fat mass (DXA).

Supplementary Table 5 Vastus lateralis thickness (ultrasound).

Supplementary Table 6 Biceps brachii thickness (ultrasound).

Supplementary Table 7 Total body water (BIS).

Supplementary Table 8 Extracellular water (BIS).

Supplementary Table 9 Intracellular water (BIS).

Supplementary Table 10 Total mood disturbance scores (POMS Questionnaire).

Supplementary Table 11 Pressure to pain threshold (Algometry).

Supplementary Table 12 Fiber count and fiber cross sectional area.

Supplementary Table 13 Training design.

Supplementary Table 14 Training volume load and repetitions in reserve.
