# Supplementary material for: Effects of Graded Whey Supplementation During Extreme-Volume Resistance Training
Source: Front Nutr. 2018 Sep 11;5:84. doi: 10.3389/fnut.2018.00084 (PMC6141782; doi:10.3389/fnut.2018.00084)
Supplement: Supplementary SDC 3 — haun_supplementary_tables.pdf—this file includes descriptive tables for each dependent variable. [file Data_Sheet_3.PDF]

SUPPLEMENTARY TABLES

Supplementary Table 1. Pre-Study Body Composition and Strength Descriptive Measurements

Variable	WP (n=10)	GWP (n=11)	MALTO (n=10)	Total (n=31)
Age (years)	21.20 ± 2.39	20.60 ± 1.51	22.10 ± 2.28	21.48 ± 2.13
Height (cm)	177.85 ± 5.60	177.75 ± 9.56	184.00 ± 7.55	179.81 ± 7.91
Weight (kg)	82.19 ± 8.69	84.51 ± 14.34	81.35 ± 10.72	82.74 ± 11.29
Total Lean Mass (kg)	63.73 ± 7.11	67.15 ± 10.90	62.06 ± 8.44	64.45 ± 9.08
Total Fat Mass (kg)	15.18 ± 4.15	13.82 ± 4.74	16.10 ± 3.55	14.94 ± 4.12
Squat 3RM (kg)	134.53 ± 21.36	135.70 ± 15.18	126.13 ± 17.25	132.24 ± 17.93
Bench Press 3RM (kg)	106.85 ± 19.47	99.82 ± 24.79	89.61 ± 11.43	98.79 ± 20.20
Stiff-Legged Deadlift 3RM (kg)	129.31 ± 33.00	140.45 ± 28.29	118.42 ± 15.75	129.75 ± 27.43
Lat Pulldown 3RM (kg)	74.41 ± 9.85	74.66 ± 17.26	68.51 ± 9.19	72.60 ± 12.73
Overhead Press 3RM (kg)	61.25 ± 10.53	55.19 ± 15.97	54.22 ± 5.18	56.83 ± 11.67

Legend: all data presented as means ± standard deviation values

Supplementary Table 2. Body Mass

Statistic	WP (n=10)	GWP (n=11)	MALTO (n=10)	TOTAL (n=31)
PRE	82.19 ± 8.69	84.51 ± 14.34	81.35 ± 10.72	82.74 ± 11.29
MID	82.32 ± 8.72	85.83 ± 14.12	83.32 ± 10.56	83.89 ± 11.18
POST	82.54 ± 8.05	86.21 ± 14.31	83.94 ± 10.84	84.29 ± 11.20
ΔMID-PRE	0.13	1.32	1.97	1.15
Effect Size (Cohen's d)	0.01	0.12	0.17	0.10
95% Confidence Intervals	-6.91 – 7.71	-5.39 – 8.03	-5.07 – 9.01	-2.83 – 5.12
ΔPOST-MID	0.22	0.38	0.62	0.41
Effect Size (Cohen's d)	0.02	0.03	0.05	0.04
95% Confidence Intervals	-6.82 – 7.26	-6.33 – 7.09	-6.42 – 7.66	-3.57 – 4.38
ΔPOST-PRE	0.35	1.70	2.59	1.55
Effect Size (Cohen's d)	0.03	0.15	0.23	0.14
95% Confidence Intervals	-6.69 – 7.39	-5.01 – 8.41	-4.45 – 9.63	-2.42 – 5.53

Legend: all PRE, MID and POST data presented as means ± standard deviation values

Supplementary Table 3. Total Lean Body Mass (DXA)

Statistic	WP (n=10)	GWP (n=11)	MALTO (n=10)	TOTAL (n=31)
PRE	63.73 ± 7.11	67.15 ± 10.90	62.06 ± 8.44	64.41 ± 8.99
MID	64.04 ± 7.29	69.15 ± 10.60	63.64 ± 8.27	65.72 ± 8.97
POST	64.95 ± 6.94	70.09 ± 10.73	64.41 ± 7.86	66.60 ± 8.85
ΔMID-PRE	0.30	1.99	1.59	1.31
Effect Size (Cohen's d)	0.03	0.22	0.18	0.15
95% Confidence Intervals	-5.21 – 5.81	-3.26 – 7.25	-3.92 – 7.09	-1.85 – 4.47
ΔPOST-MID	0.91	0.94	0.77	0.88
Effect Size (Cohen's d)	0.10	0.11	0.09	0.10
95% Confidence Intervals	-4.60 – 6.42	-4.31 – 6.19	-4.74 – 6.28	-2.28 – 4.04
ΔPOST-PRE	1.22	2.93	2.35	2.19
Effect Size (Cohen's d)	0.14	0.33	0.26	0.24
95% Confidence Intervals	-4.29 – 6.73	-2.32 – 8.19	-3.16 – 7.86	-0.97 – 5.35

Legend: all PRE, MID and POST data presented as means ± standard deviation values

Supplementary Table 4. Total Body Fat Mass (DXA)

Statistic	WP (n=10)	GWP (n=11)	MALTO (n=10)	TOTAL (n=31)
PRE	15.18 ± 4.15	13.82 ± 4.74	16.10 ± 3.55	14.99 ± 4.17
MID	15.14 ± 4.03	13.37 ± 4.42	16.51 ± 3.47	14.95 ± 4.09
POST	14.53 ± 3.91	12.82 ± 4.21	16.30 ± 3.87	14.49 ± 4.13
ΔMID-PRE	-0.04	-0.45	0.41	-0.04
Effect Size (Cohen's d)	-0.01	-0.11	0.10	-0.01
95% Confidence Intervals	-2.63 – 2.54	-2.92 – 2.01	-2.17 – 2.99	-1.51 – 1.43
ΔPOST-MID	-0.61	-0.55	-0.21	-0.46
Effect Size (Cohen's d)	-0.15	-0.13	-0.05	-0.11
95% Confidence Intervals	-3.19 – 1.98	-3.01 – 1.91	-2.79 – 2.37	-1.93 – 1.01
ΔPOST-PRE	-0.65	-1.00	0.20	-0.50
Effect Size (Cohen's d)	-0.16	-0.24	0.05	-0.12
95% Confidence Intervals	-3.23 – 1.93	-3.46 – 1.46	-2.38 – 2.78	-1.97 – 0.97

Legend: all PRE, MID and POST data presented as means ± standard deviation values

Supplementary Table 5. Vastus Lateralis Thickness (ultrasound)

Statistic	WP (n=9)	GWP (n=11)	MALTO (n=10)	TOTAL (n=30)
PRE	2.91 ± 0.51	3.07 ± 0.49	3.23 ± 0.61	3.07 ± 0.53
MID	2.87 ± 0.41	2.98 ± 0.50	3.11 ± 0.53	2.99 ± 0.47
POST	3.07 ± 0.32	3.09 ± 0.52	3.23 ± 0.49	3.14 ± 0.44
ΔMID-PRE	-0.04	-0.08	-0.12	-0.08
Effect Size (Cohen's d)	-0.08	-0.16	-0.22	-0.15
95% Confidence Intervals	-0.39 − 0.31	-0.40 − 0.23	-0.45 − 0.21	-0.27 – 0.11
ΔPOST-MID	0.20	0.10	0.12	0.15
Effect Size (Cohen's d)	0.38	0.19	0.22	0.28
95% Confidence Intervals	-0.15 − 0.55	-0.21 − 0.42	-0.21 − 0.45	-0.04 – 0.34
ΔPOST-PRE	0.16	0.02	0.00	0.07
Effect Size (Cohen's d)	0.30	0.04	0.00	0.13
95% Confidence Intervals	-0.19 − 0.51	-0.30 − 0.34	-0.33 − 0.33	-0.12 – 0.26

Legend: all PRE, MID and POST data presented as means ± standard deviation values

Supplementary Table 6. Biceps brachii Thickness (ultrasound)

Statistic	WP (n=10)	GWP (n=11)	MALTO (n=10)	TOTAL (n=31)
PRE	4.09 ± 0.51	3.87 ± 0.61	3.84 ± 0.49	3.93 ± 0.54
MID	4.22 ± 0.57	4.15 ± 0.65	4.03 ± 0.46	4.13 ± 0.56
POST	4.01 ± 0.62	4.08 ± 0.60	3.97 ± 0.44	4.02 ± 0.54
ΔMID-PRE	0.13	0.29	0.18	0.20
Effect Size (Cohen’s d)	0.25	0.53	0.34	0.37
95% Confidence Intervals	-0.20 − 0.47	-0.03 − 0.61	-0.15 − 0.52	0.01 – 0.39
ΔPOST-MID	-0.22	-0.07	-0.05	-0.11
Effect Size (Cohen’s d)	-0.40	-0.13	-0.10	-0.20
95% Confidence Intervals	-0.55 − 0.12	-0.39 − 0.25	-0.39 − 0.28	-0.30 – 0.08
ΔPOST-PRE	-0.08	0.21	0.13	0.09
Effect Size (Cohen’s d)	-0.15	0.39	0.24	0.17
95% Confidence Intervals	-0.42 − 0.25	-0.11 − 0.53	-0.21 − 0.47	-0.10 – 0.28

Legend: all PRE, MID and POST data presented as means ± standard deviation values

Supplementary Table 7. Total Body Water (BIS)

Statistic	WP (n=9)	GWP (n=11)	MALTO (n=10)	TOTAL (n=30)
PRE	50.46 ± 5.01	51.23 ± 8.65	48.13 ± 6.31	49.97 ± 6.85
MID	50.87 ± 5.87	52.12 ± 8.53	48.71 ± 5.89	50.61 ± 6.89
POST	51.38 ± 4.74	53.92 ± 8.84	49.92 ± 6.25	51.83 ± 6.95
ΔMID-PRE	0.41	0.89	0.58	0.64
Effect Size (Cohen's d)	0.06	0.13	0.09	0.09
95% Confidence Intervals	-4.03 – 4.85	-3.12 – 4.90	-3.63 – 4.79	-1.81 – 3.09
ΔPOST-MID	0.51	1.80	1.21	1.22
Effect Size (Cohen's d)	0.08	0.27	0.18	0.18
95% Confidence Intervals	-3.93 – 4.95	-2.21 – 5.81	-3.00 – 5.42	-1.23 – 3.67
ΔPOST-PRE	0.92	2.69	1.79	1.86
Effect Size (Cohen's d)	0.14	0.40	0.26	0.27
95% Confidence Intervals	-3.52 – 5.36	-1.32 – 6.70	-2.42 – 6.00	-0.59 – 4.31

Legend: all PRE, MID and POST data presented as means ± standard deviation values

Supplementary Table 8. Extracellular Water (BIS)

Statistic	WP (n=9)	GWP (n=11)	MALTO (n=10)	TOTAL (n=30)
PRE	20.00 ± 2.04	20.70 ± 3.33	19.54 ± 2.87	20.10 ± 2.79
MID	19.85 ± 2.46	21.03 ± 3.32	19.81 ± 2.76	20.27 ± 2.86
POST	20.17 ± 1.67	21.82 ± 3.66	20.44 ± 2.83	20.86 ± 2.91
ΔMID-PRE	-0.15	0.33	0.27	0.17
Effect Size (Cohen's d)	-0.05	0.12	0.10	0.06
95% Confidence Intervals	-1.98 – 1.68	-1.32 – 1.98	-1.46 – 2.00	-0.83 – 1.17
ΔPOST-MID	0.32	0.79	0.63	0.59
Effect Size (Cohen's d)	0.11	0.28	0.23	0.21
95% Confidence Intervals	-1.51 – 2.15	-0.86 – 2.44	-1.10 – 2.36	-0.41 – 1.59
ΔPOST-PRE	0.17	1.12	0.90	0.76
Effect Size (Cohen's d)	0.06	0.40	0.32	0.27
95% Confidence Intervals	-1.66 – 2.00	-0.53 – 2.77	-0.83 – 2.63	-0.24 – 1.76

Legend: all PRE, MID and POST data presented as means ± standard deviation values

Supplementary Table 9. Intracellular Water (BIS)

Statistic	WP (n=9)	GWP (n=11)	MALTO (n=10)	TOTAL (n=31)
PRE	30.45 ± 3.13	30.53 ± 5.39	28.59 ± 3.57	29.86 ± 4.18
MID	31.02 ± 3.58	31.08 ± 5.26	28.91 ± 3.29	30.34 ± 4.18
POST	31.21 ± 3.30	32.10 ± 5.20	29.49 ± 3.56	30.96 ± 4.19
ΔMID-PRE	0.57	0.55	0.32	0.48
Effect Size (Cohen's d)	0.14	0.13	0.08	0.11
95% Confidence Intervals	-2.12 – 3.26	-1.88 – 2.98	-2.23 – 2.87	-1.02 – 1.98
ΔPOST-MID	0.19	1.02	0.58	0.62
Effect Size (Cohen's d)	0.05	0.25	0.14	0.15
95% Confidence Intervals	-2.50 – 2.88	-1.41 – 3.45	-1.97 – 3.13	-0.88 – 2.12
ΔPOST-PRE	0.76	1.57	0.90	1.10
Effect Size (Cohen's d)	0.18	0.38	0.22	0.26
95% Confidence Intervals	-1.93 – 3.45	-0.86 – 4.00	-1.65 – 3.45	-0.40 – 2.60

Legend: all PRE, MID and POST data presented as means ± standard deviation values

Supplementary Table 10. Total Mood Disturbance Scores (POMS Questionnaire)

Statistic	WP (n=10)	GWP (n=11)	MALTO (n=9)	TOTAL (n=30)
PRE	85.90 ± 11.57	86.91 ± 8.99	88.78 ± 16.70	87.13 ± 12.16
MID	99.20 ± 23.00	92.64 ± 10.34	97.89 ± 23.18	96.40 ± 18.92
POST	89.50 ± 11.73	92.27 ± 8.37	99.67 ± 18.60	93.57 ± 13.42
ΔMID-PRE	13.30	5.73	9.11	9.27
Effect Size (Cohen's d)	1.10	0.47	0.75	0.76
95% Confidence Intervals	5.78 − 20.82	-1.44 − 12.90	1.18 − 17.04	4.92 – 13.62
ΔPOST-MID	-9.70	-0.36	1.78	-2.83
Effect Size (Cohen's d)	-0.80	-0.03	0.15	-0.23
95% Confidence Intervals	-17.22 − -2.18	-7.54 − 6.81	-6.51 − 9.71	-7.18 – 1.52
ΔPOST-PRE	3.60	5.36	10.89	6.43
Effect Size (Cohen's d)	0.30	0.44	0.90	0.53
95% Confidence Intervals	-3.92 − 11.12	-1.81 − 12.54	2.96 − 18.82	2.08 – 10.78

Legend: all PRE, MID and POST data presented as means ± standard deviation values

Supplementary Table 11. Pressure to Pain Threshold (Algometry)

Statistic	WP (n=10)	GWP (n=11)	MALTO (n=10)	TOTAL (n=31)
PRE	72.82 ± 10.01	65.59 ± 15.03	71.78 ± 20.56	69.40 ± 15.65
MID	66.87 ± 13.88	56.88 ± 11.68	62.40 ± 18.11	61.40 ± 14.67
POST	61.17 ± 11.24	56.73 ± 11.27	59.00 ± 17.98	59.02 ± 13.33
ΔMID-PRE	-5.95	-8.71	-9.38	-8.00
Effect Size (Cohen’s d)	-0.39	-0.57	-0.62	-0.51
95% Confidence Intervals	-15.73 − 3.47	-17.69 − 0.27	-18.80 − 0.04	-13.51 − -2.49
ΔPOST-MID	3.72	8.83	6.02	-2.38
Effect Size (Cohen’s d)	-0.38	-0.01	-0.22	-0.15
95% Confidence Intervals	-15.12 − 3.72	-9.13 − 8.83	-12.82 − 6.02	-7.89 – 3.13
ΔPOST-PRE	-11.65	-8.86	-12.78	-10.38
Effect Size (Cohen’s d)	-0.77	-0.58	-0.84	-0.66
95% Confidence Intervals	-21.07 − -2.23	-17.84 − 0.12	-22.20 − -3.36	-15.89 − -4.87

Legend: all PRE, MID and POST data presented as means ± standard deviation values

Supplementary Table 12. Fiber Count and Fiber Cross Sectional Area

Time point	Variable	WP (n=10)	GWP (n=11)	MALTO (n=10)	TOTAL (n=31)
PRE	Total Fiber Count	112.0 ± 22.3	111.0 ± 31.8	119.0 ± 28.8	114.0 ± 27.0
	Average fCSA	4038.9 ± 720.7	3957.5 ± 666.6	4092.6 ± 1184.6	4027.0 ± 853.0
	MHC II Count	56.0 ± 17.3	64.0 ± 17.4	67.0 ± 25.6	62.1 ± 20.2
	MHCII fCSA	4185.9 ± 1013.2	4053.5 ± 949.0	3952.9 ± 590.1	4063.7 ± 849.6
	MHC I Count	56.0 ± 21.9	47.0 ± 22.2	52.0 ± 17.5	51.5 ± 20.3
	MHC I fCSA	3870.7 ± 739.7	3737.7 ± 555.9	3865.7 ± 1539.0	3821.9 ± 990.8
	% MHCII	51% ± 14%	58% ± 12%	55% ± 17%	55% ± 14%
	%MHC I	49% ± 14%	42% ± 12%	45% ± 17%	45% ± 14%
MID	Total Fiber Count	114.0 ± 35.2	121.0 ± 29.3	125.0 ± 25.9	120.0 ± 30.0
	Average fCSA	3584.2 ± 782.2	3878.0 ± 503.4	3678.9 ± 436.0	3719.0 ± 584.0
	MHC II Count	56.0 ± 22.5	65.0 ± 15.7	61.0 ± 24.0	60.8 ± 20.5
	MHCII fCSA	3715.0 ± 1102.3	3940.8 ± 777.8	3735.2 ± 630.2	3801.7 ± 834.5
	MHC I Count	58.0 ± 24.6	56.*0* ± 27.7	64.0 ± 38.9	59.5 ± 30.0
	MHC I fCSA	3227.5 ± 837.2	3565.2 ± 543.2	3363.5 ± 456.8	3391.2 ± 625.7
	% MHCII	49% ± 14%	55% ± 15%	52% ± 23%	52% ± 18%
	%MHC I	51% ± 14%	45% ± 15%	48% ± 23%	48% ± 18%
POST	Total Fiber Count	100.0 ± 25.1	113.0 ± 25.7	102.0 ± 9.7	105.0 ± 22.0
	Average fCSA	4156.7 ± 586.3	4174.0 ± 944.9	4229.9 ± 516.6	4186.0 ± 694.0
	MHC II Count	46.0 ± 15.8	54.0 ± 17.9	56.0 ± 16.9	52.4 ± 16.9
	MHCII fCSA	4273.5 ± 999.6	4351.5 ± 1190.3	4251.3 ± 601.1	4294.0 ± 939.3
	MHC I Count	53.0 ± 22.7	58.0 ± 25.7	45.0 ± 16.5	52.5 ± 22.0
	MHC I fCSA	3886.7 ± 800.5	3817.8 ± 882.0	3940.4 ± 904.5	3879.5 ± 836.4
	% MHCII	47% ± 14%	55% ± 15%	52% ± 15%	51% ± 15%
	%MHC I	53% ± 14%	45% ± 15%	48% ± 15%	49% ± 15%

Legend: all data presented as means ± standard deviation values

Supplementary Table 13. Training Design

Week	# of Exercises per Session	Sets per Exercise per Week	Total Sets per Week	Repetitions per Set	Total Repetitions per Week	Intensity (% of est. 1RM)
1	4	10	40	10	400	60%
2	4	15	60	10	600	60%
3	4	20	80	10	800	60%
4	4	24	96	10	960	60%
5	4	28	112	10	1120	60%
6	4	32	128	10	1280	60%

Supplementary Table 14. Training Volume Load and Repetitions in Reserve

Week	WP (n=10)		GWP (n=11)		MALTO (n=10)		TOTAL (n=31)	
	Volume (kg)	Reps in reserve	Volume (kg)	Reps in reserve	Volume (kg)	Reps in reserve	Volume (kg)	Reps in reserve
1	28162 ± 4597	3.7 ± 1.1	27336 ± 6660	3.4 ± 1.1	25731 ± 2713	4.0 ± 0.7	27084 ± 4934	3.68 ± 0.98
2	42294 ± 6893	4.4 ± 1.2	42526 ± 7916	3.7 ± 1.0	38607 ± 4029	4.6 ± 0.7	41186 ± 6579	4.23 ± 1.02
3	56506 ± 9224	4.3 ± 1.5	51332 ± 17744	4.4 ± 1.2	51345 ± 5259	4.9 ± 0.7	53005 ± 12033	4.52 ± 1.18
4	67995 ± 11039	4.4 ± 1.5	65718 ± 13424	4.1 ± 0.9	61454 ± 6161	5.0 ± 1.2	65076 ± 10741	4.48 ± 1.24
5	79069 ± 12696	4.3 ± 1.3	73195 ± 18492	4.1 ± 1.0	72035 ± 7277	5.2 ± 1.1	74715 ± 13703	4.52 ± 1.24
6	89853 ± 14484	4.3 ± 1.6	88544 ± 15699	4.3 ± 1.4	81574 ± 8404	4.8 ± 1.2	86717 ± 13400	4.45 ± 1.36

Legend: all data presented as means ± standard deviation values
